# Human-centred artificial intelligence for mobile health sensing: challenges and opportunities

**DOI:** 10.1098/rsos.230806

**Published:** 2023-11-15

**Authors:** Ting Dang, Dimitris Spathis, Abhirup Ghosh, Cecilia Mascolo

**Affiliations:** ^1^ University of Cambridge, Cambridge, UK; ^2^ Nokia Bell Labs, Cambridge, UK; ^3^ University of Birmingham, Birmingham, UK

**Keywords:** mobile health, wearable sensing, artificial intelligence for health

## Abstract

Advances in wearable sensing and mobile computing have enabled the collection of health and well-being data outside of traditional laboratory and hospital settings, paving the way for a new era of mobile health. Meanwhile, artificial intelligence (AI) has made significant strides in various domains, demonstrating its potential to revolutionize healthcare. Devices can now diagnose diseases, predict heart irregularities and unlock the full potential of human cognition. However, the application of machine learning (ML) to mobile health sensing poses unique challenges due to noisy sensor measurements, high-dimensional data, sparse and irregular time series, heterogeneity in data, privacy concerns and resource constraints. Despite the recognition of the value of mobile sensing, leveraging these datasets has lagged behind other areas of ML. Furthermore, obtaining quality annotations and ground truth for such data is often expensive or impractical. While recent large-scale longitudinal studies have shown promise in leveraging wearable sensor data for health monitoring and prediction, they also introduce new challenges for data modelling. This paper explores the challenges and opportunities of human-centred AI for mobile health, focusing on key sensing modalities such as audio, location and activity tracking. We discuss the limitations of current approaches and propose potential solutions.

## 1. Introduction

Advances in the ways to collect and process health monitoring data can transform our society from multiple dimensions such as better access to preventive healthcare, low-cost diagnosis and long-term monitoring. An overwhelming majority of today’s medical services collect such data in laboratory settings. While an average person visits a doctor only around 5 times a year [1] these measurements provide sparse snapshots of one’s health. On the other hand, recent advances in wearable sensing and mobile computing, along with their wide adoption, have created new pathways for the collection of health and well-being data outside of laboratory and hospital settings, in a longitudinal fashion. Apart from *filling the gaps* of traditional clinical data, these devices open up new research and commercial directions for large-scale lifestyle monitoring. For example, millions of people worldwide use personal devices to track their physical activity and sleep, with increasingly more sophisticated predictive capabilities [2]. The penetration of mobile devices has introduced scale: many more individuals can now be reached and assessed. For example, in a hospital environment, mobile experience sampling enabled the collection of 11 381 survey responses over a 12-month period from 304 physicians and nurses, completed with minimal initial training [3]. Mobile sensors enable researchers to collect not only the explicit reports of the participants, but also the *context* in which these answers were provided. Indeed, a survey of 110 experience-sampling papers concluded that a total of 70 studies (63.6%) passively or actively collected sensor data from the participants’ study device [4].

At the same time, artificial intelligence (AI) has taken a huge step forward across domains ranging from object recognition in images [5], to winning against the best players in the games of Go, Atari and chess [6], or outperforming experts in breast cancer screening [7]. The widespread popularity and dramatic improvements in predictive modelling came from mature open-source scientific software libraries, easier data crowdsourcing and labelling, and the re-purposing of specialized hardware like graphics cards. In this article, we discuss how the adaptation of machine learning (ML)^1^ to mobile sensing-based human health applications is moving towards a new era of *mobile health*.

Sensors in wearable and mobile devices such as accelerometers, electrocardiograms (ECGs), global positioning systems (GPSs), gyroscopes and microphones monitor our location, sleep, steps, eating and working habits in the real world. Their continuous tracking produces huge datasets with the potential to create a holistic understanding of the most important components of our everyday health [8]. Results from a variety of fields, for example, physical activity [9], disease diagnosis [10] and mental health disorders [11], have consistently shown solid evidence that mobile health can be potentially employed for human health monitoring in the future.

Although the value of mobile sensing^2^ has been recognized, it has not been kept up with other application areas of machine learning. For instance, over the last decade devices such as Fitbit or the iPhone have been collecting multi-modal sensor data at an unprecedented temporal resolution. However, effectively leveraging these datasets presents many challenges. Some of the obstacles lie in (i) the noisy measurements of the sensor data which are hard to model due to their correlation over time or their nonlinear structure [13]; (ii) the high-dimensional raw data or engineered feature representations that are difficult to handle using conventional AI algorithms [14]; (iii) sparse and irregular-sampled time-series data; (iv) heterogeneity in different data types [15], etc. These all lead to these data being frequently overlooked for scientific and medical research. Further, obtaining quality annotations and ground truth is costly or even impossible at this granularity. The most established research and clinical practice for annotation is still based on pen-and-paper self-reports and surveys which are time-consuming and also incomplete and scarce [16].

While mobile health research data traditionally have a small number of participants and limited-sized measurements, recently there have been several large longitudinal studies like the *UK Biobank* [17], the *Apple Study* [18], the *Fenland Study* [19], *Utsureko* [20] and *EmotionSense* [21] with tens of thousands of participants using wearable sensors. These large studies have started showing their potential too; for example, an elevated resting heart rate (RHR) from over 200 000 Fitbit users was used to predict influenza-like illness in the USA [22]. However, these large studies introduced new challenges for data modelling. Statistical methods developed for small datasets often fail in these cases [23]. Additionally, how to understand and effectively model the longitudinal data over time to achieve reliable monitoring and forecasting of individuals’ health status is of great potential for more flexible and convenient health delivery. Despite these successes witnessed in mobile health, there are also inevitably a multitude of implications and limitations existing due to modelling, such as privacy concerns, human-introduced biases, practical deployment in clinical practice, and improved usability and user experience for human–computer interfaces.

**Figure 1.**
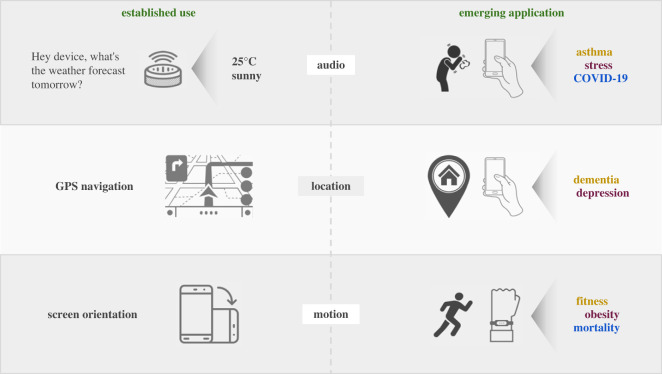
Established use of sensors and emerging applications of mobile health. An illustration of established and emerging applications that mobile sensing enables. For example, the same models that are used in speech recognition can be re-purposed for voice-based biomarker discovery (e.g. through coughing). Likewise, GPS and motion sensors can be used for early diagnosis of dementia or even all-cause mortality.

This paper discusses the challenges and opportunities of human-centred AI for mobile health, through case studies in some key mobile sensing modalities such as audio, location and activity tracking, as shown in figure 1. We particularly focus on these areas considering their popularity within both research and commercial applications. Additionally, these three modalities rely on sensors commonly found in the majority of mobile and wearable devices, unlike biosignal collection devices such as ECG chest straps or EEG headsets. Moreover, they typically involve passive sensing, minimizing the need for active user participation as opposed to camera-based vision techniques. These advantages have led to extensive research in these three modalities [24–26], suggesting their potential for future large-scale population screening and monitoring. However, we acknowledge other emerging areas such as radio sensing or biosignals in §2.5. Further, we explore the underlying limitations of current approaches, which are common across various modalities, and delve into potential solutions for these challenges.

## 2. AI for mobile health—case studies

Data analysis techniques typically are specific to the sensing modality and application at hand. However, they all follow the same general structure involving three main stages: data pre-processing, feature extraction and modelling. The pre-processing step can involve data cleaning, transformation and augmentation. The pre-processing also involves segmenting the continuous stream of data according to the specific application. The segments can simply be the sliding windows on the data stream or it can be more complex to describe and extract, for example, cough sounds detected and extracted from long noisy audio streams [27] prior to cough based modelling or trips extracted from continuous GPS traces [28]. Features are then extracted from the segments. Both human designed and learnt features have been explored and we discuss them separately for different modalities. If multiple modalities are analysed in a study, feature representations are fused [15]. ML techniques are then used for model development [29,30]. Multi-modal fusion can also be carried out at this stage, generally by fusing model outputs from different modalities [31].

Let us now take a deep dive into the three most popular sensing modalities in mobile health studies, namely, audio, location and motion sensors, and a brief look at the other modalities, including Bluetooth, Wi-Fi, etc. For each modality we start with its popular applications showing its potential to be used in mobile health applications, followed by a discussion of the common ways it is recorded, and finally detailing its mobile health applications.

### 2.1. Audio

**Audio sensing at scale.** Audio, as one of the most natural human communication signals, contains rich information. The speech and audio processing community has explored the potential of audio data for a wide range of industry applications, e.g. virtual assistants with automatic speech recognition [32], biometric system safety with speaker verification or voice antispoofing [33], etc. Recently, audio has also been analysed for health problems, including both mental and physical health, such as emotion detection [34–36], depression detection [37–39], Alzheimer’s and dementia detection [40–42], etc. Despite its wide application and great potential, most of them were carried out using data collected in the laboratory or a controlled environment. Only limited work has explored the potential of audio within the mobile health context in free-living environment. With the rapid development of mobile technologies such as smartphones, tablets and wearables, it allows for greater flexibility in data collection, processing and transmitting, offering the potential for healthcare support, delivery and intervention in a more flexible, scalable and prompt way.

### 2.1.1. Data

**Figure 2.**
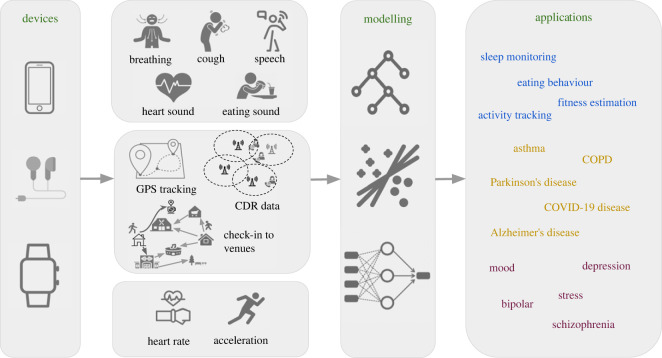
From devices to modelling and applications. An overview of the data processing pipeline for mobile health applications, including mobile and wearable devices, data types, modelling and applications. Three data types are stated: audio, location and motion sensing.

**Data types.** An overview of audio signals for health-related applications is shown in figure 2. A variety of different types of audio sounds can be easily collected from mobile phones and wearbles (e.g. smartwatch, earbuds) in daily life. *Speech*, as one of the dominant sounds, contains rich information that can infer human mental health and physical health [34,39]. Breathing and cough are two types of sounds that contain clinically relevant information and highly related to pulmonary health [10,43]. In addition, other types of sounds (e.g. eating sounds recorded via microphones) can also be informative for obesity health.

In addition to the normal airborne sound, *bone conducted sounds* collected via wearables have also been analysed for health application, which is generally collected via earables or sensors placed near neck. Chen *et al.* [44] collected sound signals from a small acoustic sensor placed at the neck to estimate heart rate (HR) and conduct heart sound classification. One other study [45] has investigated acoustics in the ear canal, measuring in-ear pulse wave velocity using heart signal as reference and the other detecting respiratory sounds [46]. Amft *et al.* [47] collected chewing sounds acquired with a microphone located inside the ear canal and used it to classify different kinds of food. Some example datasets can be found in table 1.

**Table 1.** Example datasets for audio, mobility and motion data.

	dataset	samples	participants	data types	labels
audio	Coswara [48]	2030	—	breathing, cough, speech	COVID-19, smoking status
	COVID-19 sounds [49]	53 449	36 116	breathing, cough, speech	COVID-19, smoking status, language
	RECOLA [50]	34	34	speech	arousal, valence (emotion)
	E-DAIC [39]	—	275	speech	PHQ-8 (depression)
	ADReSS challenge [40]	4076	156	voice/speech	Alzheimer’s versus non-AD, MMSE score
	chewing events [51]^a^	—	5	chewing sound	eating activities
mobility	COVID-19 Community Mobility Reports [52]	—	aggregated	aggregated data on the change in mobility trends during COVID-19	—
	mobile phone mobility data [53]	—	aggregated	aggregated movement between regions during COVID-19	—
motion	GLOBEM dataset: multi-year datasets for longitudinal human behaviour modelling generalization [54]	—	497 (700 user-years)	activity (steps, sleep), location, call logs, bluetooth, screen	depression, personality, well-being and more
	Multi-Ethnic Study of Atherosclerosis (MESA) [55]	2 200 000	1817	polysomnography (PSG), actigraphy (activity)	sleep–wake classification
	FitRec [56]	253 020	1104	heart rate, speed, GPS	workout route prediction, heart rate prediction

^a^Data for chewing events are not publicly available.

**Clinical relevance**.**** It was proven that changes in affective state and cognitive effects associated with depression have altered the mechanisms involved in speech production [57,58]. Lung disease is also expected to alter the sound generation mechanism. These changes will provide valuable information to identify between the healthy and non-healthy conditions. Some studies have analysed the significance and correlation of different audio features with mental disorder or physical disease. Though they may not be directly estimated using data collected using mobile devices, the findings should reveal certain information. Acoustic properties of speech in terms of formants and power distribution were found to be best discriminators to distinguish between depression and suicidality individuals and healthy individuals [59]. Many other studies also obtained the similar findings, and details can be found in a review paper [60]. Voice biomarkers have also been analysed for recovery from acute respiratory illness [61]. The findings suggest that jitter, shimmer and spectral contrast within certain frequency bands show consistent trends for recovering participants, and can be informative to distinguish them from healthy users as well. Slightly different conclusions might be made for different studies due to the data variability and data size, but the general conclusion of the clinical relevance of audio sounds to health and the distinguishable information between still holds.

### 2.1.2. Modelling

**Statistical analysis.** Prior to model development for mobile health applications, effective sound data types or data quality needs to be guaranteed. Dubey *et al.* [62] studied speech data collected from a smartwatch’s built-in microphone in a controlled environment, and targeted on remotely monitoring of PD patients’ speech and voices exercises to improve the communication effectiveness of speech therapy outside the clinic. The study analysed and compared speech characteristics in terms of the loudness and fundamental frequency to that collected using traditional recording methods, and showed comparable results demonstrating the quality of audio data collected through smartwatches. A continuous mobile sensing app is developed to monitor students’ life on stress, sleep, activity, mood, sociability, mental well-being and academic performance. Audio data, as one of the modalities, are recorded using smartphones. Significant correlations are obtained between conversation duration and/or frequency computed from audio data and PHQ-9 depression scores, flourishing scale and perceived stress scale (PSS) [63]. One other study [64] also analysed the audio quality of speech and respiratory sounds (e.g. cough, sneezing, etc.) collected from smartwatches in the wild, and demonstrated that the quality is good enough for humans to be able to detect speech and respiratory sounds. It also suggests the difficulty in extracting these sounds automatically from collected audio due to a wide variety of background noise. A multi-modal sensor platform for knee joint health assessment is also developed, which includes the joint sounds collected through multi-microphones [65]. The significant acoustic emissions were found to occur at similar joint angles for repeated exercises, suggesting the reliability of the audio sounds for knee activities. These statistical analysis or proof-of-concept results demonstrate the potential of audio sounds collected through different mobile devices for health monitoring.

**ML techniques**. Different ML techniques ranging from traditional methods such as support vector machines (SVMs) to deep learning techniques have been employed. Many studies using wearables such as smartwatches and earables only involve limited number of participants, where conventional ML techniques are suitable to avoid overfitting. Logistic Regression, Decision Tree and Random Forest have been employed for chewing sounds detection [47,51], eating behaviour monitoring [66], stress detection [67], etc. SVM is also a competitive technique for classification, and has been validated for stress detection [67], sleep event detection [30], cognitive impairment detection [68].

With more flexible data collection using smartphones and cloud servers, many smartphone-based studies collect audio data on a relatively large scale. Deep learning techniques have been widely employed as they generally show superior performance over traditional ML techniques with adequate and high-quality data. Different neural network structures have been validated for COVID-19 detection. A convolutional neural network (CNN) based network structure is validated using a combination of crowdsourced voice, cough and breathing sounds [10,49]. The system is developed and validated using a total of 2478 participants and yields 0.71 in terms of ROC-AUC, suggesting the potential of crowdsourced audio for COVID-19 detection. A residual network (ResNet) [29] is also employed for COVID-19 detection using cough and breathing sounds. Long short-term memory (LSTM) [69], gated recurrent units (GRUs) [70] and neural ODE processes [71] are also beneficial for COVID-19 detection and especially for COVID-19 progression prediction and forecasting, as they capture the temporal information over time in either the short term or the long term. Variations on the network structures or training strategies have also been investigated, such as the attention mechanism [72] and self-supervised learning schemes [73]. Deep learning has also been employed for lung health detection. A pre-trained CNN based network of MobileNet is employed to detect respiratory disease, and achieves an F1 score of 0.912 on healthy versus unhealthy, 0.870 on obstructive versus non-obstructive and 0.813 on COPD versus asthma classification [74]. Two types of lung sounds, namely, wheeze and crackle, are detected by semi-supervised deep learning [75].

### 2.1.3. Applications

Audio sounds contain rich health-related information and different types of audio sounds additionally reveal complementary information. It can be used for both mental and physical health detection and monitoring.

*Mental health* is important in daily life and affects a variety of human behaviours including thoughts, mood and actions. The increasing burden of mental disorders significantly impacts health and major social, human rights and economic consequences in all countries of the world. Automatic detection and monitoring of mental health could provide prompt diagnosis and reduce the burden. Depression, as one of the leading mental disorders, has been studied using audio sounds [76]. Other mental disorders have also been investigated using audio sensing, including bipolar [66], schizophrenia [77], autism [78]. Stress and mood which are highly correlated to mental health have also been studied using audio sounds [67,76].

*Physical health* studies were mainly carried out for respiratory disease and sleep health. COVID-19 has been studied using different audio sounds, including voice, breathing and cough sounds [10,29,79,80]. Breathing patterns are extracted from audio signal to monitor sleep apnoea [81], and another study explored the potential of breathing rate by leveraging smartphones for sleep monitoring, which detects sleep events including snore, cough, turn over and get up [30]. Some studies used the microphone of smartphones to quantify the flow and volume of air inspired and expired by the lungs during breathing, which reflects the degree of airflow obstruction in clinically associated pulmonary disorders such as COPD and asthma [82,83]. Heart rate and breathing rate are also estimated from in-ear microphones, and demonstrated the potential of earables for health monitoring [44,84]. This suggests new pathways for cardiovascular health monitoring using wearables. Eating behaviours are also analysed using audio collected from smartwatches and earables [47,51,85]. While audio-based applications have seen significant advancements, deploying them in real-world settings, especially for audio-based COVID-19 detection, remains challenging [86]. This primarily stems from difficulties acquiring high-quality data during the pandemic, managing variations in recording environments and devices, accounting for symptom differences associated with various virus strains, which can cause distinct audio variations, etc.

### 2.2. Mobility

Localization sensors are ubiquitous in mobile phones and wearable devices. They can measure user mobility in different spatial to temporal resolutions ranging from continuous tracking using GPS to intermittent recording of connected cell tower locations. Tracking locations and analysing mobility has contributed to many day-to-day applications, for example, traffic management and forecasting [87,88], routing individuals [89] and delivery packages [90], recommender systems [91], dynamic load balancing systems for cellular networks [92], to name a few. These applications have used techniques ranging from statistics to deep learning, and more theoretical computational geometry and algebraic topology.

Mobility naturally captures the lifestyle of individuals and thus related health information; for example, the type of restaurant a person visits reveals food habit and hence correlated health problems. In spite of its potential to unlock secrets of human health conditions, mobility has seen limited applications for mobile health primarily due to lack of large relevant datasets. Here, we first discuss the types of mobility data followed by popular features and finally health applications.

#### 2.2.1. Data types

Location of a device can be captured in various ways differing in spatial and temporal resolutions, associated privacy risks and resource requirements for the collecting device.

*GPS* sensors report the latitude and longitude of the tracked device with the aid of positioning satellites. While GPS localization is highly accurate (within a few metres), it requires clear line of sight to at least three satellites all the time; its accuracy reduces significantly in regions with obstruction. Moreover, it consumes a lot of battery power and its continuous precise recordings are often privacy invasive. Thus, popular mobile phone ecosystems like Android, iOS have recently put extra precautions for application developers to use this sensor.

*Call data record (CDR)* records the location of the base station (the cell tower a phone is connected to). It has coarse temporal resolution as the tracking generally takes place when a person uses call, messaging or data service. As a cell tower usually serves a large area its accuracy is of the order of hundreds of metres. Its coarse spatial and temporal resolution makes it less privacy invasive. Wi-Fi access points also can offer similar localization service with increased spatial and temporal resolution. While CDR is suited for outdoor localization for its wide adaptation, Wi-Fi localization is suited for indoor localization for its higher resolution and availability in indoor spaces.

*Check-in data* record when a person logs visits to venues, for example posts in social media, such as Twitter, Foursquare, Facebook, etc. Check-ins have low temporal granularity and less scaling power to a large population. However, they can capture longitudinal lifestyle with limited data size.

*Collocation* information is often more important than the location itself, for example, in the study of infectious disease spread. While this can be inferred from the above types of data, it can also be recorded directly by tracking proximity to other devices using Bluetooth or RFID.

#### 2.2.2. Features

Unlike many other sensing modalities (e.g. audio), features from mobility data are not standardized. This is primarily because complex spatio-temporal mobility data are hard to describe in a succinct unique way and different applications need to take varied perspectives.

Simple features characterizing individual trips or the spatial spread of movements are commonly used in the literature (table 2). Techniques to extract features differ based on the type of data at hand; for example, one needs to extract the interesting parts of a GPS trace prior to extracting the features, namely the stay points (where the person stayed beyond a certain duration) and the movement between stay points. Such pre-processing steps are common across applications and often offered as a framework [93].

**Table 2.** Popular features extracted from mobility data.

feature	short description
length of trips	trips are defined specific to applications, for example, movement between two stay points, or using a single mode of transport, etc.
distance from home	home location is often absent in the data for privacy reasons. It is often inferred using heuristics like the location at night
different places visited	characterized by the count of unique ones or the frequency distribution represented with entropy
radius of gyration	deviation from the centroid of the collection of places visited (*S*): $(1/\left|S\right|)\sum_{l\in S}\|l-C{\|}_{2}^{2}$, where *C* is the centroid location of *S*
entropy-based measures	entropy of the distribution of the places visited
POI-based features	variance in the type of POIs, relative frequencies, etc.

Health applications typically benefit from interpretable features to build explainable models. Healthwalks [94] builds such a framework that takes user location traces, associated points of interest and user social demographics as input and automatically generates combinations of features using a deterministic automaton (DFA). The effectiveness of the generated features is shown using an eight class disease classification. The disease categories include healthy, mental, dental, neurological, orthopaedic illness, etc.

While the above features are useful in many applications, recently researchers have been using tools from algebraic topology [95] and geometry [96] to map trajectories to points in Euclidean space to make them amenable to ML tools. Further, progress is being made for using trajectories in the ML algorithms [97,98]. However, health applications are yet to take advantage from these progresses.

#### 2.2.3. Applications

Health applications for mobility data range from individual health monitoring and diagnosis to augmenting public health decisions. In the following, we discuss different health applications that use mobility information. We divide the applications into three categories: (i) where mobility affects the disease progression in a population, (ii) where the disease changes mobility patterns in individuals that lead to digital biomarkers for diagnosis and (iii) where monitoring mobility helps in planning and managing health related resource provisioning.

The analysis pipeline differs across applications. The typical pipeline shown in figure 2 applies to the applications in the second and third category. Applications in the first category mostly use collocation data and generally use the data directly without extracting features.

**Disease spreading by mobility.** Infectious diseases such as COVID-19 and H1N1 are spread primarily by human contacts. The effect of mobility for transmitting such diseases has long been studied, for example in Spanish flu [99]. Due to COVID-19 the study of human mobility and its connection to spreading infectious diseases has surged naturally.

*Mobility-based disease spread modelling.* While traditional disease spread models like SEIR (susceptible exposed infected recovered) assume contacts between random pairs of individuals, incorporating mobility in the model increases its predictive power. For example, Schwabe *et al.* [100] proposed mobility marked Hawkes model combining Hawkes process to model the transmission dynamics of the disease and a regularized Poisson regression to incorporate mobility covariates extracted from telecommunication data. The model outperforms the other models in forecasting the infections in the future for 5–21 days in Switzerland.

*Mobility interventions.* According to the scale of movement, mobility has different effects on disease spreading, for example, fine-grained mobility within a local geographical region in terms of venues visited and number of face-to-face meetups affect the growth rate and demographic patterns of infections in that region. On the contrary, movement between countries or large geographical regions may spread a new disease or a variant to a different country or region. While both are important to study in regard to infectious diseases, fine-grained mobility is popularly captured and studied using mobile devices and is discussed in the article.

As infectious diseases spread via mobility, the most popular non-pharmaceutical interventions to reduce their spread have naturally been lockdowns. Several studies across different countries have shown their effectiveness [101,102]. Using disease simulation on aggregated inter-province movement counts in China as derived from Baidu location based services [101] has shown that the travel restrictions in Wuhan introduced on 23 January 2020 had reduced the number of cases exported internationally by 70–80% until mid February 2020. CDR data are also useful to draw similar conclusions—analysing the data for the most affected counties in the USA [102] found that the decreased mobility strongly correlates (Pearson correlation coefficients above 0.7) with decreased COVID-19 case growth rates.

Although mobility restrictions are effective in flattening the curve they immediately affected the economy and societal functions [103]. While designing the optimal lockdown policy has attracted theoretical interest [104], a more practical approach is to provide a tool for evaluating the possible effects of different intervention strategies [105]. People and venues have non-uniform effect in spreading a disease, e.g. large gatherings act as super-spreaders. Strategies to reopen an economy from lockdowns has been studied [106,107] using check-in data, Wi-Fi association logs and GPS traces. The primary conclusions are that to reduce the disease spread and retain social activities, reopening strategies need to consider the mobility of the people, for example, protect the most mobile people or restricting large gatherings.

*Mobility and other diseases.* While in the above we reviewed COVID-19 related studies for their timely relevance, mobility restrictions had been used and studied in the past, for example to stop the spread of Ebola in Sierra Leone [108], H1N1 influenza [109], etc. Also contact tracing in the context of disease spreading has long been studied [110]. Apart from the above infectious diseases, mobility has been shown to play a major role in spreading other diseases like malaria [111]—people travelling to different cities carry the parasite and infect the mosquitoes there and in turn the population.

**Mobility to aid diagnosis.** Several health problems correlate with mobility behaviour changes. Such behavioural changes are useful in predicting or diagnosing a health problem passively. This method of diagnosis is inherently less costly to deploy at large scale than traditional survey and clinical test based methods simply due to pervasive presence of mobile phones and wearable devices in the population.

*Mobility as a potential digital biomarker.* Spatial navigation patterns are affected by dementia and are considered as one of the earliest symptoms [112]. Pervasive localization sensing in personal devices and progress in related AI techniques have enabled the recent trend in research to use everyday movement data to characterize dementia. One study [113] tracked seven patients and eight healthy people for eight weeks using GPS tracking devices. It was found that the destinations for the dementia patients (with 95% accuracy) are more predictable than the control population (with 92% accuracy). Features extracted from driving trajectories are found to have potential to become digital biomarkers for Alzheimer’s disease (AD) [114]. Using the data from 75 without and 64 people with preclinical AD, the authors built a random forest classifier for identifying preclinical AD patients with F1 score as 0.82. The most important driving based features were average jerk, number of night trips and radius of gyration. Further it has been shown that Alzheimer’s patients have significantly different outdoor mobility behaviour than control population even in early stage [115]: patients explore spatially less compared to controls. A patient’s mobility patterns differ when accompanied by carers.

Individual mobility behaviour changes in fever, influenza or mental stress situations [116]. Behavioural changes are reflected in total communication, interactions with respect to time of day (e.g. late night, early morning), diversity and entropy of face-to-face interactions and movement. These are quantified using co-location and communication data tracked using Bluetooth proximity, call logs and Wi-Fi connections from the participants’ mobile phones. Finally the health status of an individual is predicted with a Bayesian network classifier, without having actual health measurements from the subjects. GPS traces also produce similar conclusions [117]—significant correlation exists between mobility trace characteristics and the depressive moods of individuals.

*Other predictive applications.* Apart from diagnostic inference at snapshots, navigation lifestyle analysis is helpful in studying longitudinal disease progression [118]. The study exploits the causal relationship between chronic diseases and daily habits captured by check-ins. The points of interest (POIs) are first embedded into 100 dimensional vectors according to their transition patterns capturing their semantic meanings. Categories of the POIs are then clustered using a Gaussian mixture model in a predefined number of clusters. The main insight here is that in a city region the distribution of POI categories is a good predictor of the distribution of progression of chronic diseases. Authors have used collaborative topic modelling to find the latent factor to model the interaction between regions and POI categories and used the same latent factorization to model the region–disease progression interaction.

It is possible to predict hospitalization using mobility behaviour [119]. The study recorded CDR data from over a thousand participants for almost two months. The hospitalization data were collected using surveys. The study used radius of gyration, distribution entropy of visited locations, area of green spaces in the neighbourhood and POI densities near the places visited. Interesting findings include that less mobile people with less visit diversity, and who visit fewer sports facilities are more likely to visit hospitals; fast food store significantly affects health of younger people.

*Analysing causality between health status and mobility.* However, one should be careful while using the correlation between mobility behaviour and health status; for example, older people visit parks more often but have worse health status than younger people. Thus a correlation analysis without considering age will lead to a negative correlation between park visits and health status. Causal relation between mobility and health status has also been analysed [120]. The proposed causal framework is based on propensity score matching. The framework is extended to health prediction models selecting causal features resulting in a robust model.

**Health resource provisioning.** Access to resources heavily affects health outcome be it the facilities for health being in the neighbourhood or critical machines in a hospital. In many such scenarios the effect can be better understood and managed by studying the locations of the facilities and their users.

*Access to outdoor facilities.* Poor physical access to health facilities causes reduced uptake of preventive health services and is likely to affect low-income households the most. A study [121] used cell tower location data from 14 million subscribers tracked for a year in rural Kenya to find the correlation between the mobility of the people, access to healthcare and uptake of two preventive healthcare interventions: childhood immunizations and antenatal care. They characterized the mobility of a person with a single measure, radius of gyration that captures both the frequencies and distances that one travels. They found that long travel times to health facilities strongly correlate with increased mobility in geographically isolated areas. Furthermore, they have shown that the mobile phone-derived measures of mobility can predict which regions are lacking preventive care.

An app was developed [122] for elderly people to locate an accessible and adequate healthcare centre that can attend to their needs. The app uses phone GPS or the cell tower location to track the user location and searches for the nearest facility from a database.

The relation between body weight and lifestyle is intuitive and has been researched using surveys and census data. Burgoine *et al.* [123] tracked school children for a week with GPS devices and found that greater school walkability was associated with significantly lower mean BMI. Greater home walkability was associated with increased BMI, as was greater school access to green space.

*Access to indoor facilities.* Indoor positioning is being used in hospitals to track medical devices and staffs for better resource management, enhancing patient experience, and repair and maintenance management [124,125]. Further indoor positioning is used to protect visually impaired, elderly people from collisions [126]. It uses RFID to identify and track passive RFID tags by analysing the received backscatter signals. By extracting the RSSI and phase profiles as fingerprints it estimates the distance to obstacles without observations of eyes and thus guiding the users to move in the domain safely.

### 2.3. Motion for physical activity and fitness

**Measuring physical activity at scale.** Large scale studies of physical activity and well-being leveraging mobile devices’ built-in accelerometers have shown promise as global physical activity surveillance tools, demonstrating, for example, inequalities across different countries after analysing data from over 700 000 people [2]. Another analysis of exercise patterns in a global social network of 1.1 million runners demonstrated that exercise is ‘contagious’, whose effect depends on gender and relative levels of activity [127]. Ten million users of a weight monitoring app were used to show that people are more likely to lose weight when they had more friends of the opposite sex [128]. Weight loss was the subject of other studies of over 1 million participants [129], which showed that power users demonstrated the greatest weight loss. The relationship between physical activity and cardiovascular disease (CVD) was studied for 50 000 people in [130], finding that lower overall activity but more frequent transitions between active and inactive periods was associated with similar CVD to higher overall activity but with fewer transitions. Mobile and wearable sensors allow for continuous and ubiquitous monitoring of an individual’s physical activity profiles, which, when combined with cardio-respiratory information, provide valuable insights into that individual’s health and fitness status [131]. Hence, the possibility of measuring individuals’ physiological characteristics in free-living conditions is of great interest for research, clinical and commercial applications. In particular, physical activity is characterized by *both* movement and the associated cardiovascular response to movement (e.g. heart rate increases after exercise and the dynamics of this increase are dictated by fitness levels [132]); thus, leveraging these two signals concurrently likely produces better representations than either signal taken in isolation. For instance, heart rate (HR) responses to exercise have been shown to be strongly predictive of CVD, coronary heart disease (CHD) and all-cause mortality [133]. In healthy individuals, HR responses to activity are defined by an increase in HR that is concurrent to the increasing intensity of the activity [134]. More recently, the Apple Watch has introduced cardio-respiratory fitness (CRF) features [135] while large population-scale studies showed that these HR responses can predict VO_2_max [136].

#### 2.3.1. Data

**Challenges in modelling wearable data.** The advent of wearable technologies has given individuals the opportunity to unobtrusively track everyday behaviour. Given the rapid growth in adoption of Internet-enabled wearable devices, sensor time-series comprise a considerable amount of user-generated data [137]. However, extracting meaning from these data can be challenging, since sensors measure low-level signals (e.g. acceleration) as opposed to the more high-level events that are usually of interest (e.g. arrhythmia, infection or obesity onset). Most wearable devices, particularly those that are wrist-worn, incorporate accelerometry sensors, which are very affordable tools to objectively study physical activity patterns [17]. However, since wearables are used in daily, unconstrained environments, activities such as drinking coffee or alcohol, as well as stress, may confound simple heuristics.

#### 2.3.2. Modeling

**Modelling wearable signals with ML.** ML models have been only recently applied to this task. To approximate VO_2_max without the need for a dynamic test, non-exercise models aim to provide a viable alternative to CRF assessment for widespread use in many healthcare settings. These are usually traditional regression models and incorporate variables like sex, age, body mass index (BMI), RHR and self-reported physical activity to infer VO_2_max [138]. However, the validity of such estimation is still much lower than what can be achieved with dynamic exercise testing [139]. Wearable devices, such as activity trackers and smartwatches, increasingly provide opportunities for non-intrusive objective monitoring of biological signals such as HR and movement during free-living, potentially enabling more precise prediction of VO_2_max without the need to conduct a specific exercise test [140]. A recent large-scale analysis used data from Fenland Study (*N* = 11 059), along with its longitudinal cohort (*N* = 2675), and a third external cohort using the UK Biobank Validation Study (*N* = 181) who underwent maximal VO_2_max testing, to show that the combination of wearables and other biomarkers as inputs to neural networks yields a strong correlation to ground truth in a holdout sample (*r* = 0.82, 95% CI: 0.80–0.83) [136]. It also detects fitness change over time (e.g. after 7 years). Notably, the model’s latent space can also be used for fitness-aware patient subtyping paving the way to scalable interventions and personalized trial recruitment. These results demonstrate the value of wearables for fitness estimation that today can be measured only with laboratory tests.

Recent advances in deep learning architectures for sequential modelling based upon wearable and mobile sensing have been used for health predictions and recommendations [141]. For example, *FitRec*, an LSTM-based approach to modelling HR and activity data for personalized fitness recommendations, was able to learn activity-specific contextual and personalized dynamics of individual user HR profiles during exercise segments [56]. This approach is helpful but requires prior segmentation of activities, which can be a constraint when applying these techniques in free-living, unconstrained conditions. Recent work using self-supervised learning has shown promise in the same data modalities such as ECG data [142,143]. Further, we recently developed a self-supervised model which exploits the multi-modal data of modern wearables to learn meaningful representations which generalize to several outcomes with transfer learning [144]. The model maps activity data to future HR responses and can be used as a feature extractor for wearable data. The value captured by the learned embeddings was showcased through strong performance at inferring physiologically meaningful variables ranging from anthropometrics to fitness, outperforming unimodal autoencoders and common biomarkers. For instance, the embeddings achieved an AUC of 0.68 for cardio-fitness prediction and an AUC of 0.80 for physical activity energy expenditure. Obtaining these outcomes in large populations can be valuable for downstream health-related inferences that would normally be costly and burdensome.

The emergence of large foundation AI models including large language models (LLMs) holds a promise to revolutionize this area. Self-supervised learning uses existing data to learn meaningful patterns by artificially creating predictive tasks such as predicting the next value or filling the gaps. In the context of health signals, these tasks can range from predicting the arrow of time, to contrastive learning and masking [145,146]. Masking, in particular, has emerged as a universal prediction task in LLMs where widely successful models such as ChatGPT are trained to predict the next word. Very recent work has demonstrated that by prompting LLMs with wearable time-series data and applying few-shot tuning, they are capable of making meaningful inferences on numerous health tasks for both clinical and wellness contexts [147].

#### 2.3.3. Applications

**Fitness as a key application.** A concept that is particularly central to physical activity is that of CRF, an important modifiable marker of cardiovascular health embodied by a strong inverse relationship with the incidence of CVD, type 2 diabetes, cancer, mortality and other adverse health outcomes [131]. Clinical evidence shows that CRF is not only potentially a stronger predictor of mortality than well-established risk factors like hypertension, type 2 diabetes, high cholesterol or smoking, but that using CRF to complement these traditional risk factors significantly improves the precision of risk prediction models for adverse CVD health outcomes [148]. Beyond its implications in medicine, CRF is frequently used in sports as an indicator of endurance capacity, having strong predictive value for other sport-related performance traits [148].

**Challenges in collecting fitness ground truth.** The *gold-standard* measure of CRF is the maximal oxygen uptake (VO_2_max), which measures the maximal rate at which an individual can consume oxygen during exercise. VO_2_max is assessed through an exercise test to exhaustion while respiratory gas exchange is measured, with the assessment only deemed a true maximal result if several test criteria are met. These criteria include levelling-off of oxygen uptake and HR and the surpassing of thresholds for the respiratory exchange ratio. This type of assessment requires trained staff and expensive laboratory settings with specialized equipment and often test criteria for maximal effort are not met [149]. Given these logistical constraints and the inherent risk of maximal exercise testing, scalability of fitness assessment in large populations has been limited, meaning relatively little is known about population levels of fitness, or their possible changes over time. Despite some promising studies which attempt to infer VO_2_max from data collected during free-living conditions, these mostly stem from small-scale cohorts with less than 50 participants and use contextual data from treadmill activity, which again limits their application in real-world contexts [150].

### 2.4. Motion for mental health

#### 2.4.1. Data

**Data and outcomes.** Mood and general mental wellbeing have been associated with several clinical outcomes. Self-reported sadness was found to be an indicator of depression [151], while self-reported happiness is linked to longevity [152], personality traits [153] and reduced mortality risk [154]. The experience sampling method (ESM) or ecological momentary assessment (EMA)—which involves asking participants to report their behaviours or environment on repeated occasions over time—has long been used as a mechanism to longitudinally assess the mental health of individuals by prompting them to report their mental state using questionnaires, traditionally administered using pen and paper and also via the Web. Psychologists have used different tools or scales to measure mood. These include the positive and negative affect schedule (PANAS) [155], a self-report questionnaire of two 10-item scales that measures both positive and negative affect; and the Affect Grid scale [156], a two-dimensional grid, where the *x*-axis indicates the feeling in terms of its positiveness or negativeness and the *y*-axis indicates its intensity. Independently of the scale used, timely and accurate mood reporting is important to anticipate clinical outcomes. To this end, smartphones and wearable devices have enabled timely delivery of experience sampling [157], allowing a near real-time detection of clinical outcomes and relapses.

#### 2.4.2. Modeling

**ML for mental health.** Regarding ML models, the majority of related literature has applied some kind of supervised learning, such as logistic regression or SVMs, which cannot capture nonlinear combinations of features. For a more extensive view of the field, we point the interested reader to a comprehensive review on ML for mental health [158]. Some recent works employ recurrent neural networks [20], feed-forward layers [159], multi-task learning [160] and autoencoders to fill in missing sensor data [161], or to learn better representations [162]. Further, binary prediction is quite common in the mood prediction literature, where mood is simplified to a binary state [21], so that, for instance, extreme depression is binned in the same class as moderate unhappiness. Since neutral mood might be uninformative and make the predictions harder, authors often omit the middle-scoring 40–60% of reports. Instead, recent work explored fine-grained mood prediction through regression and clustering [163], as well as novel multi-dimensional formulations of the Affect Grid through multi-task learning [164].

#### 2.4.3. Applications

Multiple applications of motion data for mental health have been proposed from both research and commercial ventures. For instance, *StudentLife* [63] combined sensing and self-reports to assess the impact of student workload on stress, whereas *Snapshot* [160] tracked their mood and sleep. Others focused on detecting depression by tracking medication, sleep patterns and actions [20], location [11] or keypress acceleration [165]. On a larger scale, *Utsureko* [20] and *EmotionSense* [21], two independent smartphone applications for mood monitoring through self-reports, were used by more than 24 000 and 17 000 users, respectively. On the other hand, Evidation Health, a private company, offers users money for contributing their sensor data along with self-reported surveys.

### 2.5. Other modalities

Other widely adopted sensing modalities for mobile health can be grouped into two main streams: (i) biosignal sensors and (ii) radio frequency (RF) sensing. The focus of this paper is on movement, location and audio, but we also want to acknowledge that there are other data modalities and sources that can be useful for health sensing.

**Biosignals.** The two most widely adopted sensor data types are ECG and photoplethysmography (PPG) [166–168]. ECG sensors are generally placed on the chest to measure the electrical activity generated by heart muscle contractions directly. They can collect reliable cardiac information for heart monitoring, e.g. abnormal heart rhythms, and detection of atrial fibrillation. PPG sensors are an alternative non-invasive way to monitor heart rates, which shine light on the skin surface and measure the changes in blood circulation. This information is valuable and can be used to infer the abnormality in heart rates. Due to their non-invasive and easy-to-carry nature, PPG sensors have been widely employed on smartwatches, such as the Apple Watch, to monitor cardiovascular health [169]. However, they may still have their own limitations, such as being bulky to use in daily life (e.g. ECG sensors in a chest strap), inaccurate measurement under motions (e.g. PPG sensors), sensitivity to environmental conditions, high power consumption, etc. [170]. Apart from these two types of sensors, there are also other more complex sensing modalities such as electroencephalography (EEG) and electrooculography (EOG) [171], e.g. for sleep monitoring, but they are less employed in consumer wearable and mobile devices, rather in only clinical devices that limit their employment.

**RF signals.** Bluetooth and Wi-Fi signals on mobile devices are two of the main RF signal types for mobile health. Many mobile apps have been developed using such technology to understand outbreak epidemiology, individual screening and contact tracing [172–174]. Many of these apps use Bluetooth and Bluetooth Low Energy to infer proximity between people. Multiple governments worldwide and industries have released contact tracing apps for mobile phones. For example, Singapore implemented a mobile phone application, TraceTogether, which pushes notifications to devices in close proximity as 2 m apart and sharing information [175]. Some similar apps in different countries have also been employed for COVID-19 tracing [176,177].

However, due to false negatives in detecting face-to-face contacts, digital contact tracing may not be as effective as its manual counterpart—a study [178] estimated that digital tracing alone can reduce the *R* number (average number of people infected by a single infectious person) by 44% whereas manual tracing of all contacts can reduce *R* number by 61%. Further, the apps lack in transparent quantification of individual advantage in obeying the app’s advice [179]. Moreover, people without economic privilege have a limited capacity to self-isolate at home, which undermines the effectiveness of these approaches.

Both Bluetooth and Wi-Fi signals have been widely used for indoor localization particularly for older age groups, in assisting their living quality and also the emergency response [180,181]. Wi-Fi signal is one of the key modalities for human activity recognition and gesture recognition [182]. As Wi-Fi can travel through almost any indoor corner, human body movement or gesture changes can lead to small changes in signal propagation, and thus can be used to detect activities. Recently, RF signal has also been extended for sleep posture monitoring [183], which transmits the radio signals and observes their reflections from the surroundings. It provides non-contact sleep monitoring solutions.

## 3. Implications and limitations

### 3.1. Limitation for current state of AI

**Interpretability.** A significant hurdle that hinders the wide-scale adoption of AI systems in healthcare is the lack of interpretability, making it challenging for users and medical practitioners to trust these systems. Despite previous attempts to address the challenges surrounding model interpretability, most solutions have been implemented *post hoc* (such as the attention mechanism) focusing on explanations in certain domains, such as vision [184]. For instance, while sequential deep learning can generate fairly accurate predictions when dealing with motion sequences or audio features in longitudinal time series, it remains difficult to interpret how the model makes such judgments based on historical samples. Another potential solution for enhancing interpretability is adopting uncertainty-aware modelling approaches. These enable the prediction of uncertainty associated with the final outcome predictions, thereby providing confidence levels for users to either trust or distrust the systems. One study [185] employed ensemble learning for sound-based COVID-19 detection to infer the uncertainty of the model predictions and showed that false predictions often yield higher uncertainty, enabling us to suggest to users with certainty higher than a threshold to repeat the audio test on their phones or to take clinical tests if digital diagnosis still fails. Furthermore, active learning methods could enable the selection of uncertain samples or users for further analysis such as an additional review by domain experts (physicians) [186]. Evidence also suggests that while deep learning can identify spurious correlations, it often fails to determine causation, which can be essential in a clinical context [187]. Medical practitioners need to understand the causal factors influencing outcomes rather than merely observing correlations. There is an emerging trend away from solely favouring straightforward ML systems like linear regression or SVMs to ensure explainability or overlooking interpretability in favour of performance gains using opaque deep learning models. New opportunities are blossoming in the realm of interpretability, not only encompassing previously discussed areas but also highlighting the potential of integrating domain knowledge from clinicians into modelling approaches [188].

**Adaptation and personalization.** Data are persistently accumulated over time while monitoring an individual’s health. Owing to factors like ageing or disease progression (e.g. deterioration), the attributes of such data can shift, a phenomenon referred to as the data distribution shift. Models trained on data gathered from a specific temporal cutoff may underperform in later data collection. Consequently, updating these models with new data becomes vital. Additionally, different individuals may exhibit varying symptoms or responses to the same treatment. This requires meticulous attention for systems designed around individual needs, making a case for improved personalization [189]. Such systems can adapt and cater to the heterogeneity among users, further enhancing the effectiveness of disease monitoring and healthcare interventions. Although existing methodologies like continual learning or transfer learning may present potential avenues for adaptation and personalization such as for electronic health record [190], their exploration and application in the context of mobile health remain relatively underdeveloped.

**Fairness and robustness.** Fairness ensures that algorithmic decisions do not create discriminatory or unjust results when comparing different demographics (e.g. race or sex). Real-world cases of ‘unfair’ ML algorithms abound, such as skin lesion classification models underperforming when assessing black patients, compared to white patients. At the same time, people of colour are consistently misclassified by health sensors such as oximeters as they were validated on predominantly white populations [191]. Applying ideas from ML fairness could possibly solve some of these problems; however, sensing data and models have certain particularities. For example, they typically deal with small-scale studies, proof-of-concept datasets often collected in the laboratory or in the wild. Contrary to the tabular format of such datasets, sensing data are mostly sequential in nature, with biases being harder to surface. In other words, while it is relatively straightforward to distinguish a person’s skin tone given a picture, it is much harder to do so from oximetry measurements, necessitating the collection of supplementary metadata, such as demographics. Specifically, we should consider groups relevant to a particular scenario or deployment setting. For example, in a depression screening application, gender could be relevant as a sensitive attribute due to reported gender differences in the disorder’s signals [192]. Similarly, data validation methods can facilitate fairness and robustness and, therefore, should also be applied across sensitive attributes. For example, visualization tools such as the What-If Tool [193] and FairLens [194] can help surface any potential data outliers and help correct them before they creep into models. More technically, the literature is increasingly interested in approaches that mitigate biases. Considering that most outcomes in mobile health sensing are numerical (regression tasks), Agarwal *et al.* [195] introduced two definitions of fairness in regression: *statistical parity*, which requires the prediction to be statistically independent of the sensitive attribute, and *bounded group loss*, in which the prediction error is restricted to any sensitive group to remain below some predefined threshold. Despite recent efforts within the mobile and ubiquitous computing community [196], fairness and robustness are still underexplored with regard to mobile health sensing. Robustness to distribution shifts can also be dealt with using domain adaptation or personalization techniques that are tailored to individuals and learn from their unique preferences (see previous paragraph).

### 3.2. Limitation for mobiles and wearables

**Resource constraint.** As larger models like GPT4 and other LLMs become more prevalent due to their improved capabilities, we can expect specialized foundation models tailored to healthcare applications [197]. These applications are increasingly relying on domain-specific models at the moment. This task-agnostic shift, however, presents a mounting challenge in deploying these systems on resource-limited devices such as smartphones and wearable devices, especially when considering aspects like memory capacity and battery life. Some efforts have been made on both the software and hardware to optimize deep learning inference, from model pruning and compression techniques that reduce model size and complexity to parallel network computations [198]. However, when it requires fine-tuning or retraining the models on edge devices, it becomes a challenge. This limits its applications in various contexts, e.g. personalizing the model with more new user data input, or making continual adaptation with new data. Thus, the focus is shifting towards efficient deep learning training on edge devices. Instead of transmitting local device data to a server (a process that is bandwidth-intensive and raises privacy issues), the emphasis is on distributed training methods. Typically, there is one central server paired with multiple edge devices. The breakthroughs in efficient deep learning training revolve around optimizing training update frequencies between local and server devices, determining which part of the model needs an update, and so forth [199]. Despite progress and even with advanced transformers [200], hurdles related to latency, energy consumption and compute resource management persist. Although the existing studies have not been tailored for mobile health, they share similar challenges. Hence, determining how to train and deploy deep learning models with the full potential of such resource-limited wearable devices merits thoughtful exploration.

**Data and label quality.** Very few phones and wearable devices have medical-grade sensors and are costly. The majority of devices on the market have a cheap sensor which produces noisy data or is easily interfered with by motion artefacts. This requires specific attention in data processing or model design. Proper denoising techniques before passing data for modelling or specific model structures such as multi-task learning have been validated to denoise the signals or eliminate motion artefacts. Moreover, sensor data might be missing during motions, or data are sampled at irregular time intervals in a longitudinal way. These characteristics may require additional mechanisms in AI algorithms to handle effectively. On the other hand, label quality should also be carefully examined before modelling. To make the study scalable, some labels are collected in a crowdsourced way, by self-reporting. Therefore, either further automation in the label quality check or manual check is required.

### 3.3. Limited validation of the methodology

**Limited datasets.** Data collection is time-consuming and cost-intensive. Most mobile health apps are validated only using a limited number of participants. For example, Apple’s CRF feature is publicly validated on 221 participants [135] while non-commercial methods use population-scale data from more than 11 000 participants [136]. Considering the large user bases of such products, we need to find better methods to leverage free-living with small-scale laboratory-collected data.

**Lack of validation standardization for deployment.** New drugs or treatments undergo strict certification and regulatory screenings before they become available to the public. Most commonly, the vendors conduct randomized controlled trials in order to rule out side effects and validate the efficacy thereof. In mobile health, validation is still a thorny issue. Data remain siloed within organizations and companies and most of the time treated as a ‘moat’, or a competitive advantage of each company. The few mobile health products that become available are labelled as ‘well-being’ apps so as to bypass the strict regulatory screening for medical devices. Mobile health sensing is an emerging area that should adopt more rigorous evaluation protocols from the medical AI community [201].

### 3.4. Privacy and security

Last, sensing signals from mobile phones often contain privacy-sensitive information. For example, continuously recorded audio from phones will reveal personal conversations, and mobility data can leak sensitive location visits and meetups. However, not every sensing modality poses the same threat; for example, motion data are comparatively less privacy sensitive. On the other hand, mobility data expose sensitive personal information. Moreover, security has always been a significant challenge in mobile health applications. Several studies have identified security challenges that affect the development of secure mobile health applications. For example, out of 20 000 apps from Google Play studied in [202] it was revealed that 45% of the apps rely on unencrypted communication, and as much as 23% of personal data (e.g. location information and passwords) are sent on unsecured traffic.

Both privacy and security are popular topics of research and several approaches have been proposed including anonymity frameworks [203] to more recently proposed differential privacy [204], and blockchain-based solutions [205]. More recently ML on decentralized data in terms of federated learning has shown promise to preserve privacy while building useful models out of the data [206,207]. Another popular way to mitigate privacy risks is to use on-device computation. For example, consider a location-tracking diagnostic application. A potential model using datasets acquired from multiple individuals with consent. Then the classifier can be deployed in an individual’s phone to run the inference on the user’s sensitive location data without sharing it with a third party.

While security and privacy in mobile health applications are of particular importance, we point the reader to other material that is available [208,209].

## 4. Conclusion

This paper has thoroughly examined the possibilities and challenges associated with human-centred AI for mobile health sensing, specifically focusing on three primary sensing modalities: audio, mobility and motion data. It has provided a comprehensive coverage of various aspects, ranging from data collection methods to modelling pipelines, and explored potential applications in depth. Furthermore, it has critically addressed the limitations of current approaches and proposed potential solutions, encompassing wearable devices, AI modelling and validation, while shedding light on privacy and security concerns.

The objective of this paper is to present a holistic perspective and offer valuable insights into the realm of mobile health sensing. Its aim is to enhance the understanding of human-centred AI and inspire advancements in interdisciplinary fields for future progress.

## Data Availability

This article has no additional data.

## Footnotes

1 In this paper, we use the terms ML and AI interchangeably, with ML describing the development of algorithms and statistical models such as feature engineering or unsupervised learning, and AI describing the systems or machines in general that perform tasks requiring human intelligence.

2 We define *mobile sensing* as data from connected sensors which are used to characterize behaviours related to health. Other terms used in the literature are *personal sensing*, *digital phenotyping* and *context sensing*, with different albeit overlapping connotations. We point the interested reader to the discussion in [12].
